# Excavatolide-B Enhances Contextual Memory Retrieval via Repressing the Delayed Rectifier Potassium Current in the Hippocampus

**DOI:** 10.3390/md16110405

**Published:** 2018-10-25

**Authors:** Irene Y. Huang, Yu-Luan Hsu, Chien-Chang Chen, Mei-Fang Chen, Zhi-Hong Wen, Hsien-Ting Huang, Ingrid Y. Liu

**Affiliations:** 1 Department of Molecular Biology and Human Genetics, Tzu Chi University, Hualien 970, Taiwan; yuchunirene@gate.sinica.edu.tw (I.Y.H.); to745812@gmail.com (Y.-L.H.); hsia94@gms.tcu.edu.tw (H.-T.H.); 2 Institute of Biomedical Sciences, Academia Sinica, 128, Academia road, Section 2, Nangang, Taipei 115, Taiwan; ccchen@ibms.sinica.edu.tw; 3 Cardiovascular and Metabolomics Research Center, Department of Medical Research, Buddhist Tzu Chi General Hospital, Hualien 970, Taiwan; mfchen@mail.tcu.edu.tw; 4 Department of Marine Biotechnology and Resources, National Sun Yat-sen University, Kaohsiung 804, Taiwan; wzh@mail.nsysu.edu.tw; 5 Institute of Medical Sciences, Tzu Chi University, Hualien 970, Taiwan

**Keywords:** coral, excavatolide-B, *Ca_v_3.2^−/−^* mice, T-type calcium channel, potassium current, long-term potentiation, autism spectrum disorder, absence epilepsy, memory

## Abstract

Memory retrieval dysfunction is a symptom of schizophrenia, autism spectrum disorder (ASD), and absence epilepsy (AE), as well as an early sign of Alzheimer’s disease. To date, few drugs have been reported to enhance memory retrieval. Here, we found that a coral-derived natural product, excavatolide-B (Exc-B), enhances contextual memory retrieval in both wild-type and *Ca_v_3.2^−/−^* mice via repressing the delayed rectifier potassium current, thus lowering the threshold for action potential initiation and enhancing induction of long-term potentiation (LTP). The human *CACNA1H* gene encodes a T-type calcium channel (Ca_v_3.2), and its mutation is associated with schizophrenia, ASD, and AE, which are all characterized by abnormal memory function. Our previous publication demonstrated that *Ca_v_3.2^−/−^* mice exhibit impaired contextual-associated memory retrieval, whilst their retrieval of spatial memory and auditory cued memory remain intact. The effect of Exc-B on enhancing the retrieval of context-associated memory provides a hope for novel drug development.

## 1. Introduction

A deficit in memory retrieval is a common symptom observed in patients with brain disorders, including childhood absence epilepsy (CAE) [1], autism spectrum disorder (ASD) [2,3], schizophrenia [4,5], and particularly Alzheimer’s disease (AD) [6]. Currently, all prescribed medications approved to treat symptoms of memory dysfunction in early to moderate stages are drugs that function as cholinesterase inhibitors, including donepezil, rivastigmine, and galantamine. These drugs can only delay or slow the worsening of the memory retrieval deficit but cannot cure the symptom [7]. In addition, effectiveness of these drugs varies among individuals, and side effects including nausea, vomiting, loss of appetite, and increased frequency of bowel movements are often reported [8]. Therefore, development of a drug for the prevention or reversal of memory loss with fewer side effects remains an important theme in memory dysfunction research.

Drugs with novel or alternative mechanisms of action are the best sources for the selection and development of memory drug(s) with minimum harmful side effects. More than 50% of drugs in clinical use are derived from natural products [9]. Among natural products, marine organisms provide a tremendous source and potential for drug discovery. Living in a challenging oceanic environment, marine organisms produce compounds that defend against various biotic and abiotic stresses. Many of them have been studied for their bioactivities, including antiviral, anticoagulant, and anti-inflammatory effects [10]. Most compounds from marine sources are extracted from either coral reefs or sponges [11]. Excavatolide-B (Exc-B), a chemical compound categorized in the class of diterpenes, was discovered by Sheu et el. from a *Formosan gorgonian* soft coral *Briareum excavatum* [12]. Exc-B is known to be effective in increasing the L-type calcium current and decreasing the late sodium current, Na^+^/Ca2^+^ exchange current, transient outward current, and delayed rectifier potassium current in LPS-treated atrial myocytes [13].

The effect of Exc-B on the central nervous system (CNS) has not yet been studied. Given its effect on calcium, sodium, and potassium currents, which are important for the alteration of membrane potential and function of memory retrieval, we thus investigate the effect of Exc-B on memory retrieval. Here, we demonstrate that Exc-B is effective in enhancing contextual memory retrieval via repressing the outward potassium current and enhancing the induction of early long-term potentiation (LTP). We further validated the effect of Exc-B on *Ca_v_3.2^−/−^* mice, which exhibit a phenotype of impaired contextual memory retrieval [14]. The *Cacna1h* gene encodes the α1 subunit of the voltage-gated Ca_v_3.2 T-type (transient) calcium channel, which plays important roles in various organs, including the heart and the brain [15]. Voltage-gated calcium channels are composed of several subunits: α1, α2δ, β, and γ subunits. Of these subunits, the α1 subunit determines the channel characteristics [14,15,16]. Many studies have revealed the involvement of Ca_v_3.2 T-channel in the pathogenesis of cardiac hypertrophy and cardiac fibrosis [17]. The human *CACNA1H* gene is also associated with schizophrenia [18], ASD [2,16], and absence seizures (AS) [19]. These diseases are all characterized by memory deficits to a certain extent. Injection of Exc-B before retrieval of contextual memory reversed the defective phenotype of the *Ca_v_3.2^−/−^* mice. This research suggests that Exc-B is a novel and potential natural product that acts as a memory enhancer to treat symptoms of memory retrieval deficits associated with various brain disorders.

## 2. Results

### 2.1. Exc-B Repressed Consolidation, Enhanced Retrieval of Contextual Memory, and Did Not Affect Tone Memory

**Figure 1:**
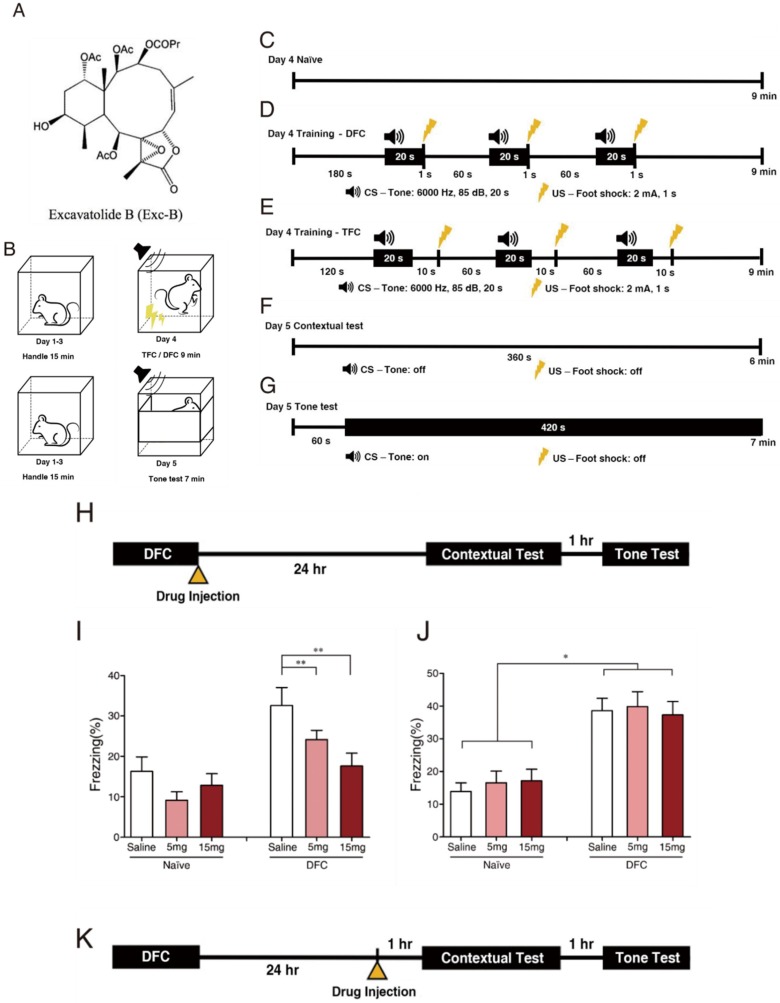

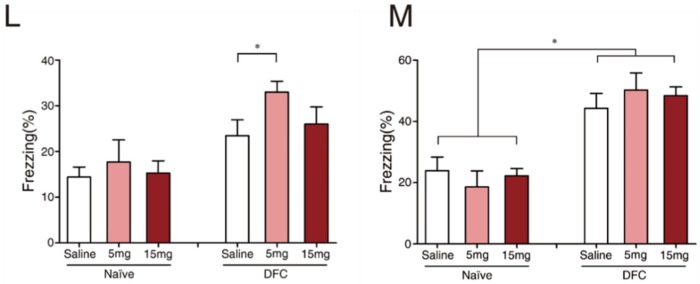
The effect of the Exc-B on contextual memory and tone testing in different concentration. The structure of Excavatolide-B (Exc-B) (**A**) Day schedule of fear conditioning training and testings’ (**B**). Fear conditioning time line (Day 4) for naïve control (**C**), delay fear conditioning (DFC, (**D**)), and trace fear conditioning (TFC, (**E**)). Testing time line to context (**F**), and Tone (**G**). DFC experimental process and the time point of Exc-B injection for investigating its effect on memory consolidation (**H**). Exc-B injection right after training resulted in significant reduction of freezing to context in a concentration-dependent trend (**I**). No significant effect of the Exc-B on consolidation of tone memory (**J**). DFC experimental process and the time point of Exc-B injection for investigating its effect on memory retrieval (**K**). Freezing % to context was significantly increased in the 5 mg/kg Exc-B-treated group (**L**). Freezing % to tone indicated no difference among groups (**M**). (* indicates *p* ≤ 0.05; ** indicates *p* ≤ 0.01).

To assess the effect of Exc-B on different stages of fear memory formation, we injected the compound before consolidation (Figure 1H) and retrieval (Figure 1K) of fear memory. The results showed that Exc-B significantly repressed the consolidation of contextual memory (Figure 1I) in a concentration-dependent manner, whereas it did not affect that of tone memory (Figure 1J). Exc-B injection before memory retrieval significantly enhanced the retrieval of contextual memory at a concentration of 5 mg/kg (Figure 1L), whilst no effect was recorded on the retrieval of tone memory (Figure 1M). The normal locomotor activity of the mice after drug injection suggests that the freezing behavior was due to the animal’s fear conditioning response and not drug administration (Supplementary Figure S1).

### 2.2. Exc-B Injection Reversed Impaired Memory Retrieval to the Context in Ca_v_3.2^−/−^ Mice

**Figure 2:**
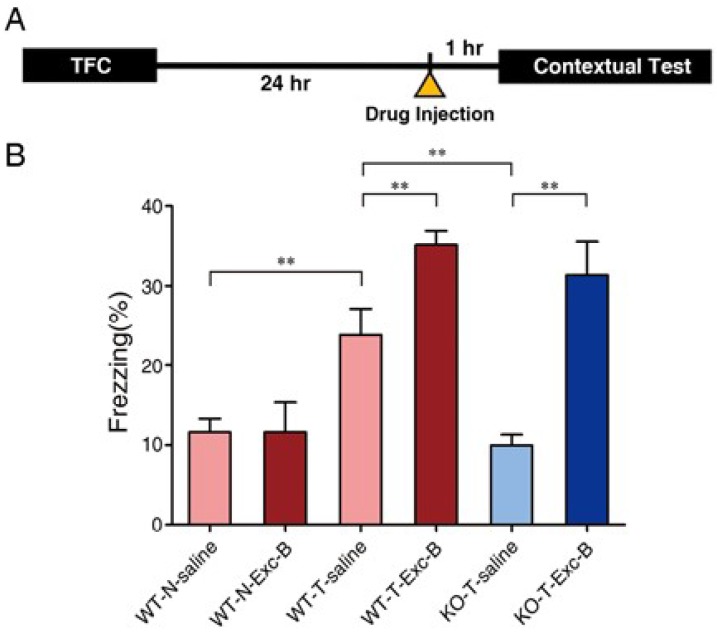

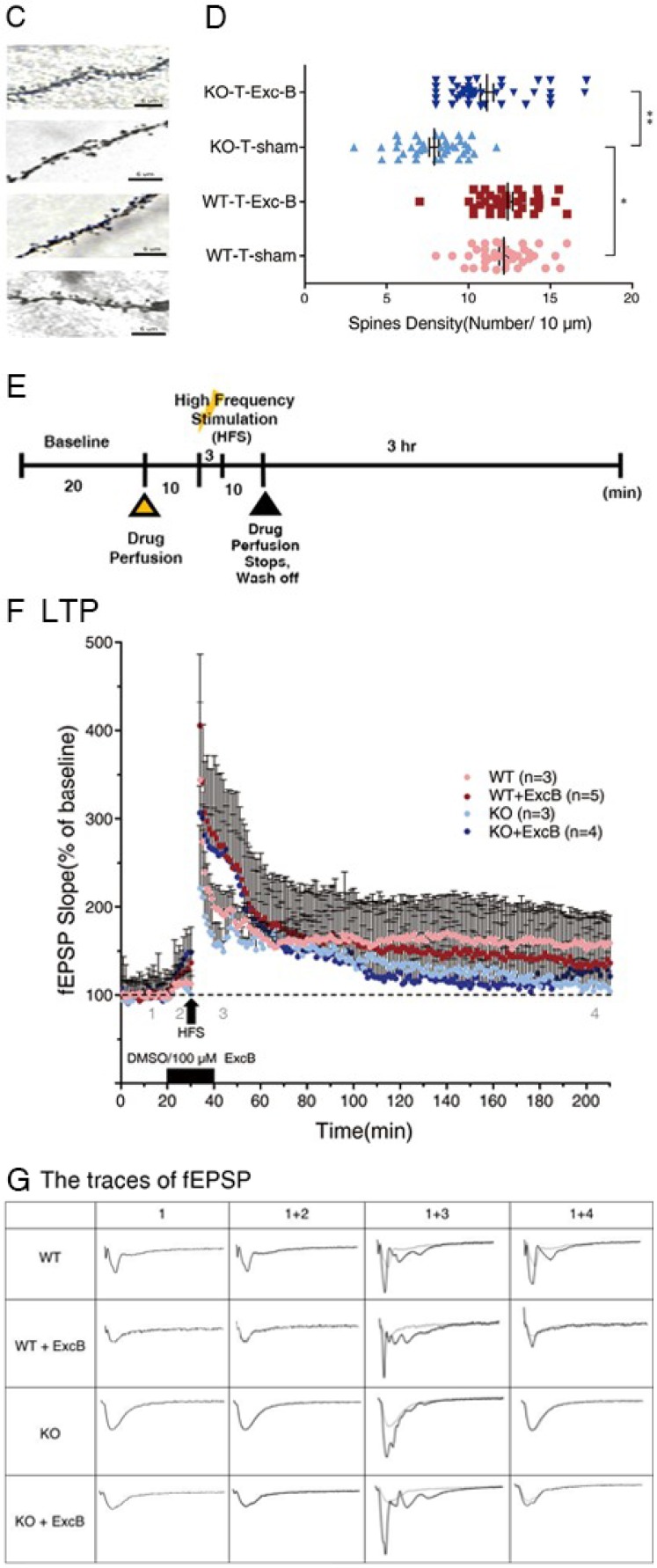

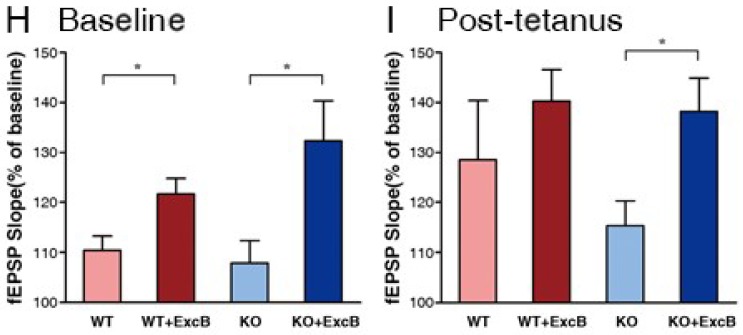
The effect of Exc-B on memory retrieval and long-term potentiation of the wild-type and the Ca_v_3.2 knockout (KO) mice. The Exc-B effect on memory retrieval of the wild type and the Ca_v_3.2 knockout (KO) mice. Twenty-four hours after TFC, one hour before contextual test, the Exc-B was injected to both WT and KO mice to evaluate its effect on memory retrieval (**A**). The Exc-B injection significantly enhanced contextual memory retrieval of the wild-type mice and reversed the impaired memory retrieval to context of the *Ca_v_3.2^−/−^* (KO) mice (**B**). The Exc-B injection significantly increased spine density of the *Ca_v_3.2^−/−^* (KO) mice after contextual memory retrieval (**C**,**D**). Experimental process and drug perfusion time line for long-term potentiation (LTP) recording (**E**). Results are represented as percentage of pre-HFS baseline, as normalized field excitatory postsynaptic potential (fEPSP) slope in the wild-type and *Ca_v_3.2^−/−^* brain slices perfused with/ without 100 μM Exc-B (**F**). The traces of fEPSP: “1” indicates the end of 10 min in baseline recording; “2” indicates the baseline recording with Exc-B for 10 min; “3” indicates the beginning of 10 min after high frequency stimulation (HFS) (with Exc-B bath), also called post-tetanus; “4” indicates the last 10 min of recording (Exc-B has been washed off) (**G**). Baseline was significantly enhanced after the Exc-B treatment in both wild-type and the *Ca_v_3.2^−/−^* (KO) groups (**H**). The amplitude of post-tetanus was significantly enhanced in the *Ca_v_3.2^−/−^* brain slices with the Exc-B treatment (**I**). (* indicates *p* ≤ 0.05; ** indicates *p* ≤ 0.01).

According to our previous publication, *Ca_v_3.2^−/−^* mice show impaired retrieval of contextual memory [14]. Since Exc-B at 5 mg/kg significantly enhanced the retrieval of contextual memory, we injected it into the *Ca_v_3.2^−/−^* mice intraperitoneally 1 h before retrieval of contextual memory (Figure 2A) to validate its effect. Results showed that at a concentration of 5 mg/kg, Exc-B enhanced contextual memory retrieval in the wild type (WT) mice by approximately 10%, and reversed the defective contextual memory retrieval phenotype of the *Ca_v_3.2^−/−^* mice to the level similar to wild-type, Exc-B-treated mice (Figure 2B).

### 2.3. Exc-B Increased Dendritic Spine Density in Ca_v_3.2^−/−^ Mice

Dendritic spines are responsible for neuronal plasticity, and the spine density indicates the strength of memory formation [20]. To further validate the effect of Exc-B on memory performance in the *Ca_v_3.2^−/−^* mice at the cellular level, we measured the dendritic spine density in the CA1 region. The results demonstrated a significant increase in the apical dendritic spine density in the Exc-B-treated *Ca_v_3.2^−/−^* mice (Figure 2C). Quantification analysis indicates that the dendritic spine density in the Exc-B-treated *Ca_v_3.2^−/−^* group is similar to that of the WT group (Figure 2D). This provides cellular morphology data to support behavioral results.

### 2.4. Early Long-Term Potentiation (LTP) Was Enhanced with Exc-B Injection

To examine the effect of Exc-B on synaptic plasticity, we measured LTP through ex vivo electrophysiological recording in the CA1 region of hippocampal slices bathed with 100 μM Exc-B. The baseline was recorded for 20 min, and then, Exc-B was applied to the slice for 20 min. Ten minutes after Exc-B application, LTP was induced with high-frequency stimulation (HFS) (Figure 2E). The slope of fEPSP along the time span (Figure 2F) and traces of fEPSP (Figure 2G) indicate that the baseline was enhanced by 10% in wild-type mice (*n* = 5) and 22% in *Ca_v_3.2^−/−^* mice (*n* = 4) (Figure 2H) after Exc-B application. In addition, the post-tetanic amplitude in the Exc-B-treated *Ca_v_3.2^−/−^* group was significantly enhanced by 64% compared with that in the drug-free group (*n* = 4, *p* ≤ 0.05), whilst Exc-B did not affect the post-tetanic amplitude of wild-type mice (Figure 2F,I). In contrast, 100 μM Exc-B reduced the late phase LTP in both wild-type and *Ca_v_3.2^−/−^* hippocampal slices (Figure 2F). The results demonstrated that after Exc-B administration, baseline and post-tetanic synaptic transmission of the *Ca_v_3.2^−/−^* mice were enhanced, whilst late-phase LTP was attenuated.

### 2.5. Exc-B Inhibited the Potassium Current Amplitude in Hippocampal Neurons

**Figure 3:**
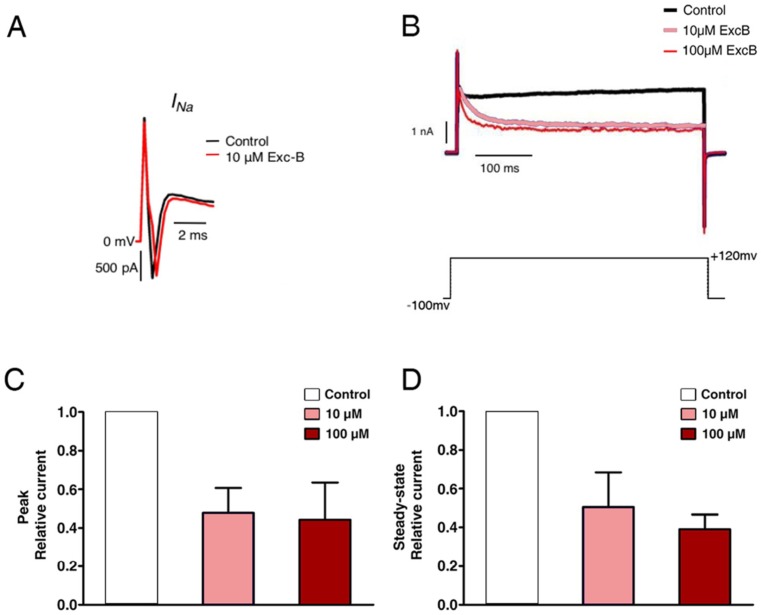

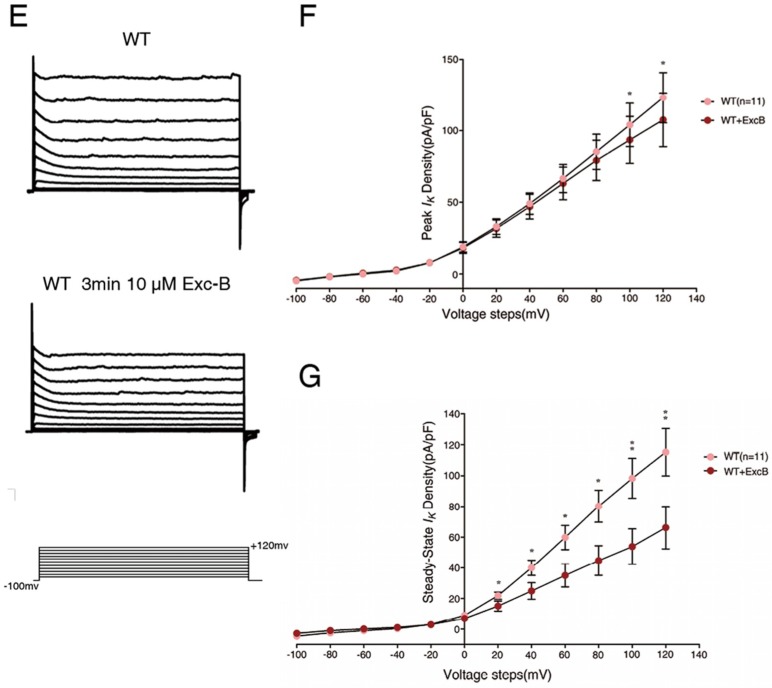
The effect of the Exc-B on potassium current. The Exc-B is effective on inhibiting outward potassium current, but not inhibiting sodium current (**A**). The Exc-B inhibited outward potassium current and exhibited saturation effect in a concentration-dependent manner (**B**). The 100 μM Exc-B and 10 μM Exc-B exhibited no significant difference regarding the effect on peak (**C**) (*n* = 7, *p* = 0.35) and steady-state (**D**) potassium current (*n* = 7, *p* = 0.07). Potassium current in wild-type hippocampal neuronal cells was inhibited after 10 μM Exc-B application by a step from −100 mV to +120 mV (**E**). Current-voltage (I–V) curve illustrated the effect of Exc-B on peak current. The Exc-B application did not change the peak current (**F**). Current-voltage (I–V) curve illustrated the effect of Exc-B on steady-state current. The Exc-B significantly inhibited steady-state potassium current by 45 ± 4% at membrane voltage stepping from −100 mV to +120 mV (**G**). (* indicates *p* ≤ 0.05, ** indicates *p* ≤ 0.005).

To further investigate the mechanism of the Exc-B effect on synaptic plasticity, we examined what type(s) of channels/currents were affected. According to whole-cell current recordings in primary hippocampal neurons derived from postnatal wild-type mice (postnatal day 1–3, P1–P3), Exc-B administration inhibited the potassium current but not the sodium current (*n* = 7) (Figure 3A). Exc-B inhibited the outward potassium current and exhibited a saturation effect in a concentration-dependent manner (Figure 3B). Quantitative analysis revealed that the potassium outward current observed at the beginning of a 20 ms step from −100 mV to +120 mV was inhibited to 58 ± 3% of the control level by 10 μM Exc-B (*n* = 7), and 59 ± 4% of the control level by 100 μM Exc-B (*n* = 7) (*p* < 10^−3^ for all concentrations relative to control; *p* = 0.35 between 10 and 100 μM) (Figure 3C). The current traces indicated the presence of a steady-state outward potassium current at the end of the 20 ms voltage step, in which delayed rectifiers but not A-type potassium channels (IA) are involved. The outward potassium current at the end of the 20 ms step from −100 mV to +120 mV was also reduced to 49 ± 10% of the control level by the addition of 10 μM Exc-B (*n* = 7) and 53 ± 10% of the control level by Exc-B application (*n* = 7) (*p* < 0.05 for all concentrations relative to control; *p* = 0.07 between 10 and 100 μM) (Figure 3D).

To further confirm this phenomenon, the voltage-activated potassium current was measured from hippocampal neurons of wild-type mice. Figure 3E illustrates the traces of the potassium current recorded from wild-types before and after Exc-B application. Additionally, Figure 3F demonstrates the current-voltage curve (I–V curve) of the peak current, which corresponded to the A-type potassium current (*I_A_*) for the first 20 ms from the baseline. The peak outward current from −100 mV to +120 mV within the first 20 ms was suppressed 10 ± 2%, with application of 10 μM Exc-B (*n* = 10, *p* ≤ 0.05) (Figure 3F). During the steady-state, the *I_k_* was much more stable within the period during the last 20 ms. Exc-B remarkably inhibited the steady-state potassium current by 45 ± 4% when the membrane voltage was stepped from −100 mV to +120 mV (*n* = 10, *p* ≤ 0.01) (Figure 3G). Taken together, the inhibitory effect of Exc-B on the steady-state potassium current was more robust than that on the peak-state current.

### 2.6. Exc-B Reduced the Increased Potassium Current in the Hippocampal Neurons of the Ca_v_3.2^−/−^ Mice

**Figure 4:**
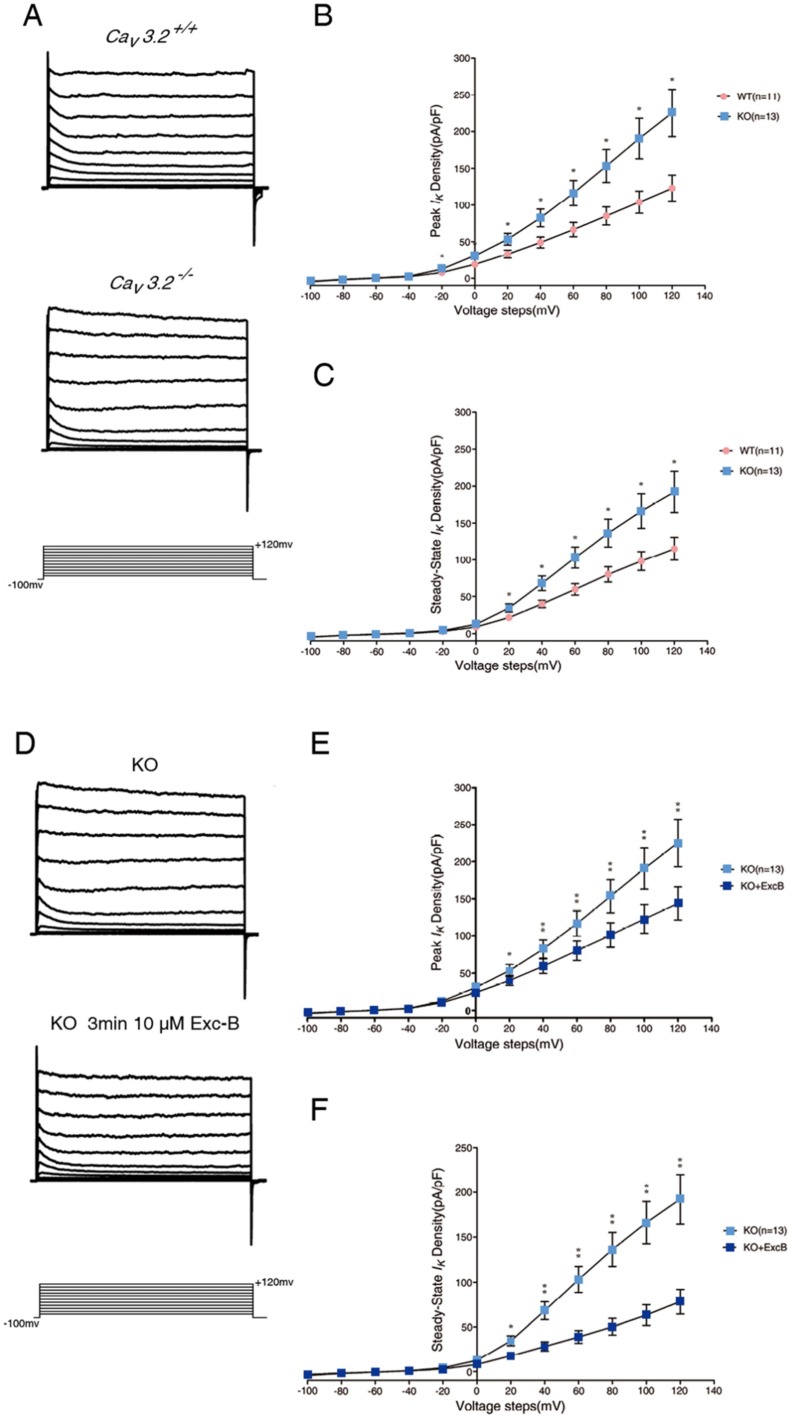

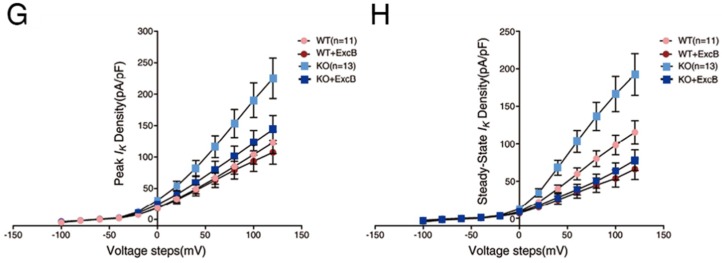
The effect of Exc-B on reversing potassium current phenotype of *Ca_v_3.2^−/−^* (KO). Potassium current in the wild-type and *Ca_v_3.2^−/−^* (KO) hippocampal neurons by voltage step from −100 mV to +120 mV (**A**). In the *Ca_v_3.2^−/−^* (KO) hippocampal neurons, both peak potassium current (**B**) and steady-state current (**C**) were significantly higher than that of the wild-types. Application of the Exc-B significantly reduced potassium current in the *Ca_v_3.2^−/−^* hippocampal neuronal cells by steps from −100 mV to +120 mV (**D**). Both peak potassium current (**E**) and steady-state current (**F**) in *Ca_v_3.2^−/−^* (KO) hippocampal neurons was significantly inhibited by 10 μM Exc-B. The Exc-B effectively reduced higher potassium current in the *Ca_v_3.2^−/−^* neurons to wild-type level on both peak current (**G**) and steady-state outward current (**H**). (* indicates *p* ≤ 0.05, ** indicates *p* ≤ 0.005).

To investigate whether Exc-B reversed the impaired memory retrieval of the *Ca_v_3.2^−/−^* mice, and is mediated via the regulation of potassium currents, we recorded voltage-dependent potassium currents from *Ca_v_3.2^−/−^* hippocampal neurons. The potassium currents recorded from the *Ca_v_3.2^−/−^* hippocampal neurons were larger than those recorded from the WT neurons (Figure 4A). The peak potassium current was 75 ± 5% (Figure 4B) and the steady-state current was 57 ± 4% larger (Figure 4C) in the neurons of the *Ca_v_3.2^−/−^* neurons than in the wild-type neurons (*n* = 8, *p* ≤ 0.05). Next, we examined whether the Exc-B can reverse the enhanced potassium currents in the *Ca_v_3.2^−/−^* neurons. We applied 10 μM of Exc-B to the hippocampal neurons, followed by patch clamp recording. Our results showed that 10 μM Exc-B reduced the potassium current by 45 ± 6% in the *Ca_v_3.2^−/−^* neurons (Figure 4D). The intensity of both the peak (Figure 4E) and steady-state (Figure 4F) potassium current was significantly reduced by the Exc-B and was reversed to the wild-type level (Figure 4G,H).

## 3. Discussion

The present research elucidates the electrophysiological mechanism of the effect of Exc-B on enhancing memory retrieval. To the best of our knowledge, this is the first study to demonstrate that the coral-derived compound Exc-B is effective in enhancing memory retrieval. This is also the first study to report that loss function of the Ca_v_3.2 T channel results in an increased potassium current, which can be reversed by Exc-B treatment. Acute ex vivo administration of Exc-B to hippocampal slices reduces the outward potassium current, increases baseline synaptic transmission and post-tetanic potentiation (PTP), and reduces late-phase LTP. The results are correlated with our behavioral data: Exc-B facilitates the induction of LTP (baseline to PTP) and corresponds to memory retrieval enhancement; Exc-B decreases the maintenance of LTP (after PTP), and is relevant to the effect of Exc-B on the repression of memory consolidation.

The effect of Exc-B on LTP is similar to that of methamphetamine (METH), which also increases baseline transmission but suppresses the late phase of LTP [21]. Interestingly, the NMDA receptor antagonist dl-2-amino-5-phosphonovaleric acid (DL-APV) does not block METH-induced baseline synaptic transmission [21]. This evidence indicates that increased baseline transmission is not dependent on NMDA receptors and the corresponding release of monoamine neurotransmitters (including dopamine, serotonin, and adrenaline) does not require action potential induction [21]. Monoamine neurotransmitters can alter synaptic transmission via the D1 like-receptor [22]. Given that Exc-B demonstrates a similar effect as METH on LTP, Exc-B may also function through a similar molecular mechanism. The dopamine D1/D2-like receptor is reported to contribute to memory retrieval [23] by modulating several molecules that are critical for memory retrieval. Activation of the D1-like dopamine receptor inhibits the K_v_1-mediated D-current and further regulates the waveforms of propagating action potentials in axons [24]. K_v_1 potassium channels can also modulate dopamine release and D2 receptor function [25]. To understand the molecular mechanism of the effect of Exc-B on enhancing memory retrieval, further studies may target the dopaminergic pathway and related molecules in the hippocampus.

Our results demonstrate that Exc-B repressed consolidation but enhanced fear memory retrieval to conditioned context. On the other hand, it has been reported that low-dose METH (1 mg/kg) treatment on mice facilitates consolidation but not spatial memory retrieval, as tested with the Morris water maze (MWM) protocol [26]. In this research, we did not use the MWM to investigate the effect of Exc-B on spatial memory. The discrepancy between the effects of METH and Exc-B on memory consolidation and retrieval may be due to different molecular pathways and brain regions involved in the two distinct types (MWM and FC) of memory. Further experiments are necessary to understand the effect of Exc-B on the formation of spatial memory.

**Figure 5:**
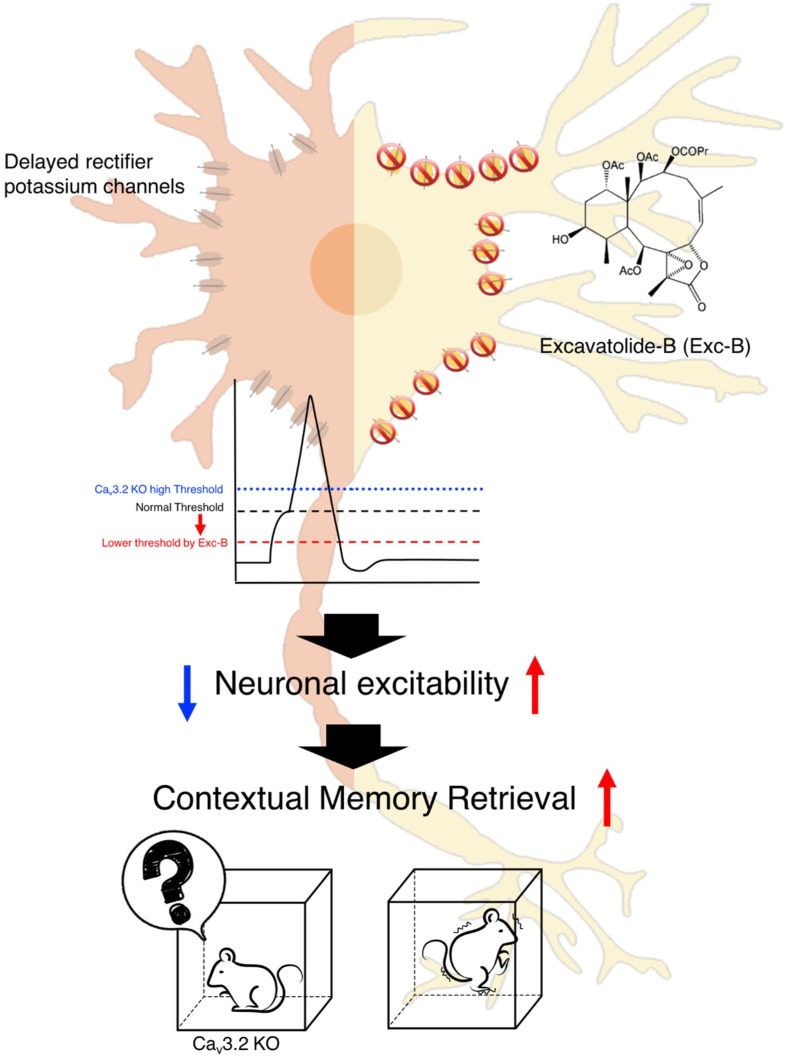
The mechanism of the Exc-B effect on memory retrieval. The mechanism of the Exc-B effect on memory retrieval. The Exc-B inhibits delayed rectifier potassium current and decreases the firing threshold of action potential. Faster neuronal excitability facilitates to enhance contextual memory retrieval. In the *Ca_v_3.2^−/−^* (KO) mice, due to higher potassium current, the threshold for firing action potential is increased (blue line) compared to wild-type (black line). The Exc-B application inhibits potassium current of the *Ca_v_3.2^−/−^* (KO) mice, thus lowers the threshold for initiating action potential (red line) and LTP, subsequently rescuing impaired phenotype of contextual memory retrieval.

In the present study, *Ca_v_3.2^−/−^* hippocampal neuronal cells produce larger outward potassium currents than wild-type neurons, and Exc-B restores the enhanced potassium current to the wild-type level. Upregulated potassium current by application of potassium channel agonist results in amnesia in mice [27]. In addition, an increase in the delayed rectifier potassium current is found in the hippocampal neurons of scopolamine-induced memory-deficient rats [28]. Scopolamine is a classical inhibitor of the muscarinic acetylcholine receptor [28]. The two reports support our finding that impaired memory retrieval exhibited by the Ca_v_3.2 knockout mice was associated with a larger potassium current. Potassium currents are involved in the modulation of neuronal excitability via modulation of spike frequency, amplitude, and time precision. This indicates that overexpression of potassium current may affect memory processing. Although we report that the potassium current appears to be larger in the Ca_v_3.2 knockout mice, which bear the loss-function T-type calcium channel, it is not clear yet which type of potassium channel is involved. It has been shown that induction of long-term potentiation of intrinsic excitability (LTP-IE) occurs along with a reduction in the D-type potassium current. In addition, the LTP-IE is dependent on the back-propagating action potentials and intact distal apical dendrites in the hippocampal CA3 pyramidal cells [29]. Further experiments are required to identify particular type(s) of potassium channel affected by loss of the Ca_v_3.2 T-type calcium channel and inhibited by Exc-B. According to our results, we hypothesize that Exc-B can enhance calcium influx and suppress the delayed rectifier potassium current through a Ca_v_3.2 T channel-dependent signaling pathway. In turn, the decreased potassium current would reduce the threshold for action potential induction to further regulate intrinsic excitability. In Figure 5, we illustrate the hypothesized model for the effect of Exc-B on altering the relationship between the Ca_v_3.2 T channel, potassium channels, and memory retrieval. In this research, though we did not demonstrate whether Exc-B can pass the blood-brain-barrier (BBB) or not, we assume it did target the brain regions and show its effect on memory retrieval after intraperitoneal injection with its molecular weight of 595.6 Da and liposoluble feature [30]. Further pharmacological and biochemical analyses are required to prove that Exc-B does enter the BBB, and to verify which of its metabolite is effective.

The Ca_v_3.2 mutation is known to be associated with schizophrenia [4,5,18], ASD [2,3,16], and AE [1,18]. Patients affected with one of these three neurodegenerative diseases/epilepsy syndrome often suffer from cognitive impairments, especially impaired memory retrieval [1,31,32,33,34]. Moreover, people affected with Alzheimer’s disease (AD) are considered unable to form new memories and defective in retrieving old memories [6,35]. The D-type potassium channel, K_v_1.3, is highly expressed by microglia in patients with AD [36], and upregulation of the A-type potassium channel is found in AD neurons [37]. Amyloid β protein (Aβ1-40), one of the main pathogenic factors of AD, has been shown to increase delayed rectifier and A-type potassium current. Potassium current upregulation is involved in the Aβ peptide-induced apoptotic pathway and results in neuronal death [38,39]. Currently, the drugs for treating AD, including galantamine and bis (3)-tacrine, are acetylcholinesterase inhibitors with the ability to suppress the delayed rectifier potassium current [40,41]. The common side effects of galantamine are gastrointestinal symptoms, such as nausea, vomiting, diarrhea, and anorexia [42]. The application of bis(n)-tacrine produces hepatotoxic effects in patients [43]. Therefore, development of new drugs with greater efficacy and fewer side effects for the treatment of memory dysfunction associated with AD and various brain disorders is an urgent necessity. Our research indicates that Exc-B may be a promising natural compound for treating memory retrieval deficit of these diseases.

## 4. Materials and Methods

### 4.1. Ethics Statement

All protocols used in this study have been reviewed and approved by the Institutional Animal Care and Use Committee of Tzu Chi University (TCU, #104105) and follow the Taiwan Ministry of Science and Technology guidelines for ethical treatment of animals.

### 4.2. Animals

C57BL/6J wild-type male mice, originally provided by the National Laboratory Animal Centre were purchased and maintained undisturbed in the Laboratory Animal Centre of TCU until the behavioral tasks were performed. The homozygous knock-out mice (*Ca_v_3.2^−/−^*) were generated as described previously in Reference [44]. Amplification of exon 6 was performed using PCR to genotype the individual mice. The primers used for the amplification of exon 6 were as follows:

Primer#1—5′-ATTCAAGGGCTTCCACAGGGTA-3′,

Primer#2—5′-CATCTCAGGGCCTCTGGACCAC-3′,

Primer#3—5′-GCTAAAGCGCATGCTCCAGACTG-3′.

All mice used in the experiments were between 8 and 12 weeks old. Animals were housed in individual plastic and metal cages with ad libitum access to food and water under a constant 12-h light/dark cycle. All experiments on mice and behavioral analysis were performed in a double-blinded manner.

### 4.3. Drug Administration

The structure of Exc-B is illustrated in Figure 1A. It was produced at Professor Ping-Jyun Sung’s laboratory in Taiwan’s National Museum of Marine Biology and Aquarium. It is a liposoluble compound with molecular weight of 595.6 Da.

### 4.4. Locomotor and Social Behavior Test

Wild-type mice were divided into two groups: (a) Sham controls, and (b) intraperitoneal (i.p.) injection with 5 mg/kg Exc-B. Mice received behavioral training one hour after Exc-B injection.

#### 4.4.1. Open Field Test

The mice were placed in an open chamber (50 cm × 50 cm × 50 cm) with no cue or stimulus for 15 min and were allowed to freely move around. A tracking device (Track Mot, Drinstrument, Taiwan) was used to measure locomotor activity.

#### 4.4.2. Fear Conditioning (FC)

The animals then underwent FC and memory tests. The wild-type mice were handled in the conditioning chamber for 15 min per day for three days (Figure 1B). On the 4th day, naïve controls were placed inside the conditioning chamber without presentation of tone or foot shock for 9 min (Figure 1C). Trained groups received three delayed FC (DFC) trials composed of a 20 s tone (CS) followed by 1 s foot shock (US), with an intertraining interval of 1 min (Figure 1D). For trace fear conditioning (TFC), the mice were given three TFC trials composed of CS followed by a 10 s temporal interval and US (Figure 1E). The Exc-B was injected into mice immediately after the training to investigate its effect on consolidation. Twenty-four hours later (the 5th day), the mice were placed into the same conditioning chamber for 6 min without being given tone or foot shock for contextual testing (Figure 1F). One hour after the contextual test, mice were placed in a different chamber. No tone or shock was given during the first min, followed by 6 min of tone exposure (Figure 1G). To study the effect of Exc-B on memory retrieval, the Exc-B was injected in a separate group of mice 24 h after FC. One hour after injection, contextual test followed by tone test was performed. Twenty-four hours later (the 5th day), mice received injection of Exc-B followed by contextual test (Figure 1F). The FC experiments were video recorded, and the freezing behavior was analyzed using FreezeScan 1.0 software (Clever Sys, Inc., Reston, VA, USA).

### 4.5. Golgi Staining and Quantification of Dendritic Spine Density

Mouse brains were removed with perfusion, and the whole brain was incubated in Golgi-Cox solution for 14 days in darkness. The Golgi-cox solution was prepared as described previously in Reference [45]. Two weeks later, the brains were transferred and incubated in 30% sucrose until they sank. The brains were sectioned into 60-µm slices and staining procedure was applied as described previously [45]. Dendritic spine density was calculated using ImageJ (downloaded from National Institute of Health, Bethesda, MD, USA, https://imagej.nih.gov/ij/download.html). For each group, 3 brains were perfused and dissected; for each brain, 5 sections were examined, and for each section, the dendritic spine density was measured and calculated in 10 neurons (5 from the left hippocampus and 5 from the right hippocampus) using the following formula: Dendritic spine density = total number of spines/length of dendrite.

### 4.6. Primary Neuronal Cell Culture

Primary neuronal cell culture was set up according to the methods previously described in Reference [46]. Hippocampal neuronal cells were acutely dissociated from postnatal day 1–3 C57BL/6J mice. Whole brains were removed and placed in the dissection medium to collect hippocampi. Hippocampi were trypsinized at 37 °C in a water bath for 15 min and then mixed gently with DNase (Sigma-Aldrich, St. Louis, MO, USA) solution for 3 min. Then the hippocampi were triturated by fire-polished 1000 μL plastic tips with plating medium. Approximately 40,000–60,000 cells were dropped onto a 10-mm coverslip precoated with 0.01 mg/mL poly-l-lysine. Maintenance medium (96% neurobasal medium, B-27 supplement, 200 mM glutamine, penicillin/streptomycin with 0.01% neuron growth factor (NGF, Gibco Thermo Fisher, Waltham, MA, USA)) was added to each well.

### 4.7. Whole-Cell Patch Recording

Primary neuronal cells were recorded on day in vitro (DIV) 3 to 8. Recordings were performed using an Axopatch 200B Microelectrode Amplifier (Axon Instrument, Union City, CA, USA). Signals were filtered at 1 kHz and digitized at 2 kHz by Digidata 1322A (Axon Instruments, Union City, CA, USA) using pCLAMP 10.2 software (Molecular Device, San Jose, CA, USA). Glass capillaries (1.5 mm OD; World Precision Instruments, Sarasota, FL, USA) were pulled on a microelectrode P-97 puller (Sutter Instrument, Novato CA, USA) and filled with internal solution. Coverslips with dissociated cells were perfused with external physiological solution (Tyrode’s solution) at pH 7.4. The resistance of the glass capillaries was 5–8 MΩ. Tetrodotoxin (TTX) (2 μM) was applied in external solution to block sodium currents. The Exc-B was prepared as a 10 mM stock solution in 100% DMSO and diluted in the external solution.

### 4.8. LTP Recording

Four- to six-week-old wild-type and *Ca_v_3.2^−/−^* mice were anesthetized with isoflurane and sacrificed by guillotine. Whole brains were immediately removed and placed into ice-cold artificial cerebrospinal fluid (ACSF). The hippocampi were isolated quickly and placed in the groove of a 3% agarose block with the CA1 facing outside and the anterior part facing upward. The posterior 1/5 of the hippocampus was trimmed off and blocked to produce a flat edge. The flat interface was glued to the metal chamber of a vibrating microtome (Microslicer DTK-1000, Dosaka EM Co. Ltd., Kyoto, Japan). The hippocampi were bathed in oxygenated ACSF and cut into 450 μm transverse slices. The hippocampal slices were collected and placed in incubated boxes with ACSF at room temperature (27 °C) for 90–120 min. Slices were transferred to a recording chamber and perfused with ACSF containing 0.1 mM picrotoxin at a speed of 2–3 mL/min. Two bipolar stainless-steel microelectrodes (Frederick Haer Company, Bowdoinham, ME, USA) and glass pipettes were pulled on a micropipette puller (PC-10 Needle Puller, Narishige, Japan) filled with 3 M NaCl, and were positioned at the stratum radiatum of the CA1 for recording the excitatory postsynaptic field potentials (fEPSPs). The stimulation intensity was adjusted between 0–80 μA for each slice, so that the fEPSPs were elicited to approximately 50% of the maximal response. Baseline fEPSPs were evoked every 15 s for 30 min followed by high-frequency stimulation (HFS), including 3 trials of 100 pulses at 100 Hz for 60 s. Then, fEPSPs were elicited every 15 s for an additional 180 min. The sham solution (ACSF + DMSO) and 100 μM Exc-B were applied at the end of the 10 min baseline recording period until 10 min after HFS application. Exc-B was dissolved in DMSO first and then diluted in ACSF with a final DMSO concentration of 0.1%. Recordings were amplified using an Axon Multiclamp 700B amplifier. All signals were filtered at 1 kHz and digitized at 10 kHz by a CED Micro Power 1401 mKII interface (Cambridge Electronic Design, Cambridge, UK) using Signal software. The downward slope of fEPSPs was measured.

### 4.9. Statistical Analysis

The statistical analysis of the behavior data was performed using SigmaPlot. Behavioral data were presented as the mean ± SD, and electrophysiological data were presented as the mean ± SEM. For comparisons between more than two groups, one-way ANOVA was used, and a *p* value < 0.05 was considered statistically significant. For comparison between two groups, Student’s *t*-test was used. The graphs were plotted with GraphPad software Prism 6.0.

## Supplementary Materials

The following are available online at http://www.mdpi.com/1660-3397/16/11/405/s1, Figure S1: Locomotor activity of mice before and after Exc-B injection is similar. Moving distance of mice for a testing period of 15 min before and after Exc-B injection is similar, indicating that locomotor activity is not affected.
