# Supplementary material for: Excavatolide-B Enhances Contextual Memory Retrieval via Repressing the Delayed Rectifier Potassium Current in the Hippocampus
Source: Mar Drugs. 2018 Oct 25;16(11):405. doi: 10.3390/md16110405 (PMC6266063; doi:10.3390/md16110405)
Supplement: Supplementary file 1 [file marinedrugs-16-00405-s001.pdf]

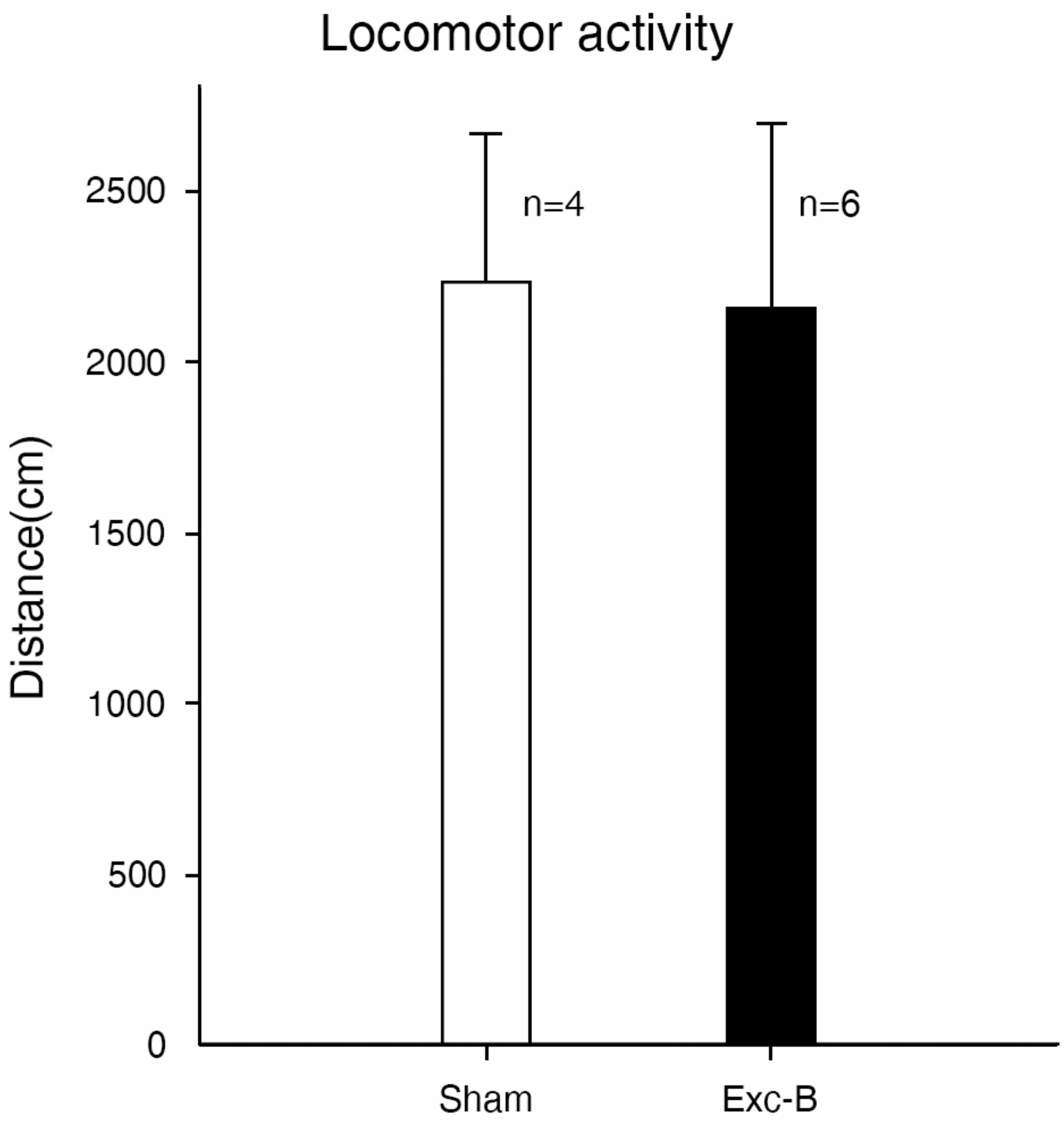

Figure S1. Locomotor activity of mice before and after Exc-B injection is similar. Moving distance of mice for a testing period of 15 min before and after Exc-B injection is similar, indicating that locomotor activity is not affected.
